# Dual right coronary artery: an unconventional presentation from Pakistan

**DOI:** 10.11604/pamj.2020.36.221.21307

**Published:** 2020-07-28

**Authors:** Jahanzeb Malik, Nismat Javed

**Affiliations:** 1 Department of Cardiology, Rawalpindi Institute of Cardiology, Rawalpindi, Pakistan,; 2 Shifa College of Medicine, Shifa Tameer-e-Millat University, Islamabad, Pakistan

**Keywords:** Coronary artery, dual, RCA, intervention

## Abstract

There is a significant variation in the mapping of epicardial coronary arteries. This variation may present as different anomalies. These anomalies are usually asymptomatic and can be present at birth. Some might become symptomatic during adulthood and subsequently, result in sudden death. The anomalies, in turn, present a challenge to clinicians during therapeutic intervention. Therefore, there is a growing need to have knowledge about the various forms of the coronary artery anomalies. In our case report, we present the case of a patient who had one such anomaly and provide a hint at one strategy that was used to tackle the challenge.

## Introduction

A coronary anomaly is defined as an irregularity in the course, structure or origin of the epicardial coronary arteries. Various studies show the frequency of about 0.2%-1.3% [1]. Dual right coronary artery (RCA) is one of the rare forms of coronary aberrancy. Mostly it is benign, but sometimes it presents a challenge during coronary intervention. In this case we reported an atypical double RCA which emerged after a conventional coronary angiogram.

## Patient and observation

**Figure 1:**
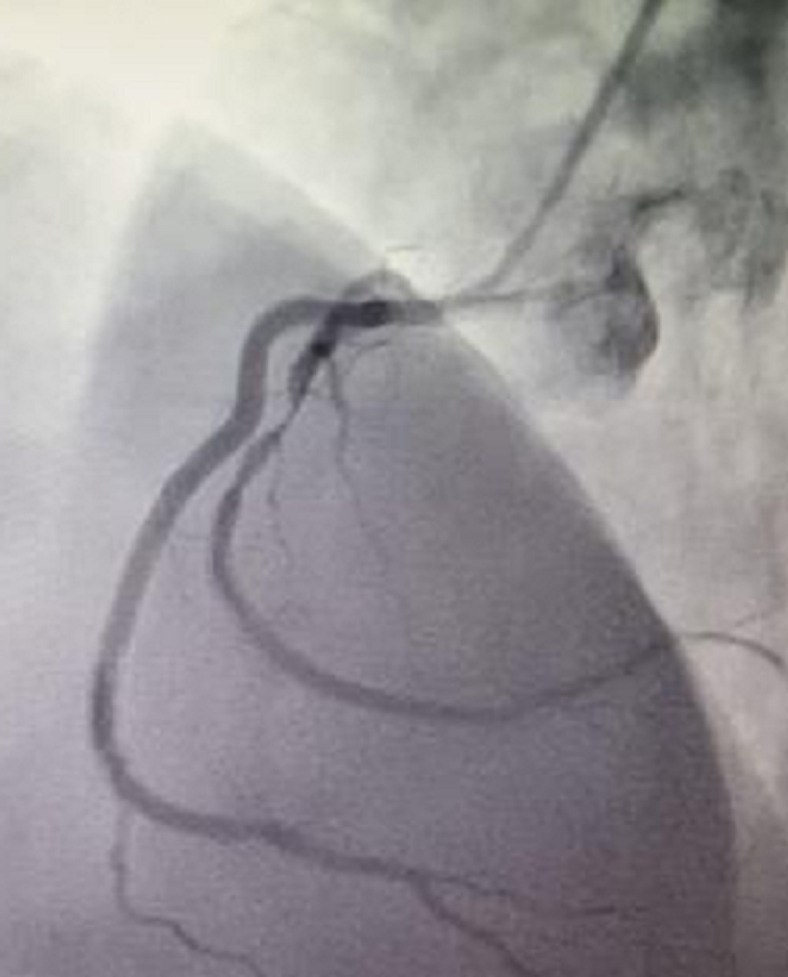
dual RCA emerging from the main stalk with diseased second vessel

**Figure 2:**
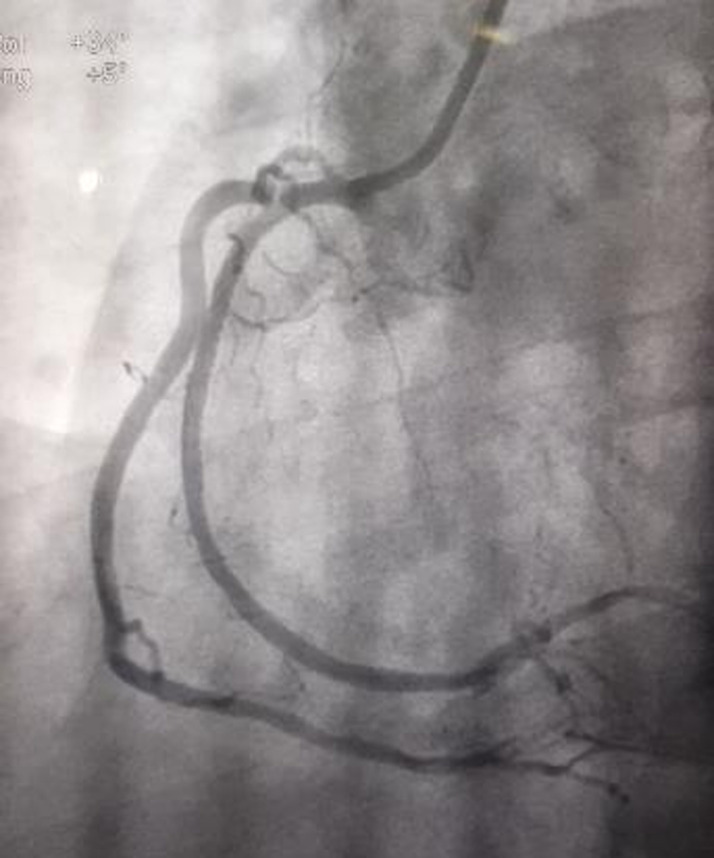
dual right coronary artery after angioplasty

**Figure 3:**
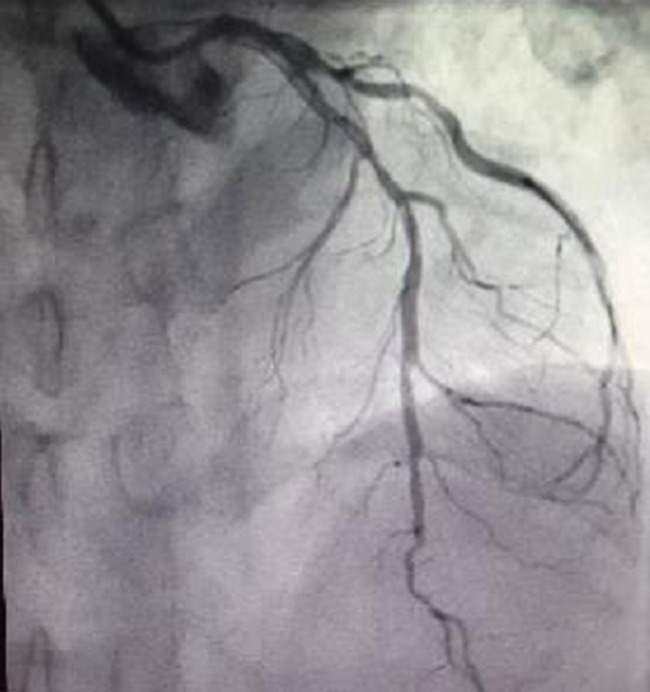
left anterior descending artery (LAD) with a critical diffuse disease from proximal throughout mid-course

A 52-year-old male patient presented with five months history of effort angina of Canadian cardiovascular society class II. He was a known type II diabetic for the past three years controlled by oral hypoglycemic medicines. Other history was non-significant. Electrocardiography and echocardiography showed no abnormalities but his exercise tolerance test (ETT) was positive for ischemia with significant ST sagging in inferior leads. Hematology and biochemistry profiles were normal. He was booked for a coronary angiogram which showed two right coronary arteries (RCA) arising from a main stem. The second artery had a critical disease in its proximal course with 99% stenosis (Figure 1, Figure 2 and Figure 3). The major obtuse marginal (OM) branch had a critical disease in proximal segment and atrioventricular branch was totally occluded. Angioplasty of the diseased vessels with three drug-eluting stents was proceeded and the patient admitted in post-cath ward for the procedural twenty-four-hour observation. Next day he was discharged on dual-antiplatelet drugs.

## Discussion

Coronary anomalies have multiplicity of presentations which portray diverse manifestations and pathophysiological mechanisms. They are congenital, but very few of them are of any sequela. Being customarily benign, they can have a serious clinical outcome like myocardial infarction, arrhythmias, congestive cardiac failure and sudden death [2]. In contrast to left coronary system, which is mainly subdivided into LAD and left circumflex (LCX), giving off many small tributaries, right coronary artery is isolated and branches off into posterior left ventricular (PLV) and posterior descending artery (PDA) in its distal course and seldom gives any substantial branches in its proximal and mid segments. Statistically, double RCA is a very unusual and rare coronary anomaly. It has been reported in less than 30 times [3-5]. However, their recognition and classification at the time of coronary angiogram is very important to quantify and plan for intervention, if required. The true definition and appropriate diagnosis of this anomaly is controversial in literature reviews. A study concluded that it is not always easy to differentiate dual RCA from high splitting of PLV branch. In reality it is a split RCA, wrongly labelled as dual RCA [6]. Another study has shown that during coronary angiography, right anterior oblique (RAO) view can help in differentiating the dual RCA from the large right ventricular artery [7]. Disregarding this fact, it is a rare anomaly and very few cases have been reported in the literature.

## Conclusion

Despite the controversial definition of dual RCA, RAO view can help in the disparity of the various aberrancies. Based on the previous reports, the clinical course of the variation is amiable, except when atherosclerosis is involved, which can present a challenge during coronary intervention. We recommend using a non-selected cine when cannulating the RCA for the first time so that the two arteries can be established originating from the same or the different ostium.

## References

[1] Ripley DP, Saha A, Teis A, Uddin A, Bijsterveld P, Kidambi A (2014). The distribution and prognosis of anomalous coronary arteries identified by cardiovascular magnetic resonance: 15 year experience from two tertiary centres. J Cardiovasc Magn Reson.

[2] Yuksel S, Meric M, Soylu K, Gulel O, Zengin H, Demircan S (2013). The primary anomalies of coronary artery origin and course: a coronary angiographic analysis of 16,573 patients. Exp Clin Cardiol.

[3] Akcay A, Koroglu S, Kaya H, Koleoglu M, Acar G (2010). An unusual appearance of double right coronary artery. Cardiol Res Pract.

[4] Sari I, Kizilkan N, Sucu M, Davutoglu V, Ozer O, Soydinc S (2008). Double right coronary artery: report of two cases and review of the literature. Int J Cardiol.

[5] Rohit M, Bagga S, Talwar KK (2008). Double right coronary artery with acute inferior wall myocardial infarction. J Invasive Cardiol.

[6] Natarajan M, Kumarguru BN, Biligi DS, Raghupathi AR (2015). Double right coronary artery originating from separate ostia: a report of two cases. N Am J Med Sci.

[7] Altun A, Akdemir O, Erdogan O, Ozbay G (2002). An interesting diagnostic dilemma: double right coronary artery or high take off of a large right ventricular branch. Int J Cardiol.

